# Antipsychotic effects on anthropometric outcomes in anorexia nervosa: a retrospective chart review of hospitalized children and adolescents

**DOI:** 10.1186/s40337-023-00862-4

**Published:** 2023-09-06

**Authors:** Bettina Frank, Sabine Arnold, Charlotte Jaite, Christoph U. Correll

**Affiliations:** 1 Department of Child and Adolescent Psychiatry, Psychosomatic Medicine and Psychotherapy, Charité-Universitaetsmedizin Berlin, Campus Virchow, Corporate Member of Freie Universitaet Berlin, Humboldt Universitaet zu Berlin, and Berlin Institute of Health, Augustenburger Platz 1, 13353 Berlin, Germany; 2 Department of Clinical Psychology and Psychotherapy in Childhood and Adolescence, University of Hildesheim, Hildesheim, Germany; 3 Department of Psychiatry, The Zucker Hillside Hospital, Northwell Health, Glen Oaks, NY USA; 4 Department of Psychiatry and Molecular Medicine, Zucker School of Medicine at Hofstra/Northwell, Hempstead, NY USA

**Keywords:** Anorexia nervosa, Children, Adolescents, Youth, Weight gain, Antipsychotics, Correlates

## Abstract

**Background:**

Off-label antipsychotic use is not uncommon in youth with anorexia nervosa (AN), aiming to enhance suboptimal weight restoration, yet its efficacy remains debated, especially in youth.

**Methods:**

Retrospective chart review of consecutively admitted inpatients (ages 8–18 years) with restricting/binge-purge AN, comparing youth with versus without antipsychotic treatment regarding baseline factors, treatment, and anthropometric outcome characteristics including all patients and matched subgroups. Matched subsamples were also compared regarding faster versus slower weight change (median split). Furthermore, within-subject analyses compared weight gain trajectories before versus after antipsychotic use in antipsychotic-treated youth. These results were then compared in a pre-/post design with the matched control group without antipsychotic treatment, using the mean duration until antipsychotic use in the antipsychotic-treated group as the dividing timeline, controlling for a potential order effect, in that later rather than earlier antipsychotic treatment for AN may be more successful.

**Results:**

Of 294 youth with AN (median age = 15.2 (interquartile range = 14.0, 16.6) years, females = 96.6%, restricting subtype = 81.0%, hospitalization duration = 98.2 ± 43.2 days), 44 (15.0%) underwent 52 antipsychotic trials (olanzapine = 63.5%). In multivariable analyses, antipsychotic use was independently associated with younger age, childhood physical abuse history, comorbid borderline personality traits, and lower pre-antipsychotic weight gain (*p* < 0.0001). In unmatched groups, antipsychotic-treated versus non-treated youth had significantly lower discharge anthropometric parameters, longer inpatient treatment, and lower weight change/week (*p* < 0.001–*p* = 0.005), without significant differences between olanzapine and non-olanzapine antipsychotics (*p* = 0.27–0.44). Non-significant antipsychotic effects on weight outcomes were confirmed in (1) matched subgroups of antipsychotic-treated versus non-treated youth (n = 43 each), (2) youth with faster versus slower weight gain (n = 21 vs. n = 22), and (3) antipsychotic-treated youth when comparing weight change before versus after antipsychotic use (n = 31). Moreover, in antipsychotic-treated youth, weight change/week remained significantly lower versus matched non-antipsychotic-treated youth (n = 31) both before (*p* = 0.053) and after (*p* = 0.006) the median time (5 weeks) until antipsychotic use.

**Conclusions:**

In this naturalistic study, clinician's antipsychotic choice, given to a more severely ill subgroup of adolescents with AN, did not significantly improve overall worse weight change trajectories during inpatient treatment, even in matched subgroups. Randomized trials in individuals reflecting real-world samples are needed to evaluate the utility of antipsychotic treatment in youth with AN.

**Supplementary Information:**

The online version contains supplementary material available at 10.1186/s40337-023-00862-4.

## Abstract (plain language summary)

Anorexia nervosa is one of the psychiatric diseases with the highest risk of death in adolescence. People with anorexia nervosa are often severely underweight out of fear of being too “fat”. Treatment is difficult. Clinicians not infrequently use off-label antipsychotic medications to improve weight gain in anorexia nervosa. Unfortunately, currently, there is little evidence of efficacy for antipsychotics, especially in children and adolescents. We examined the files of youth (8–18 years old) who were treated as inpatients between 1992 and 2015. Altogether, 44 (15%) of the 294 youth were prescribed antipsychotics, with antipsychotic use increasing over time (1992 = 0% to 2015 = 20%). Of all antipsychotic drugs, olanzapine was given most frequently (64%). Patients who received antipsychotic medications were younger and sicker than patients without antipsychotics. Also, patients prescribed antipsychotics gained less weight per week before starting antipsychotics. They also gained less weight than patients not receiving antipsychotics. Despite being treated longer as inpatients, antipsychotic-treated youth had lower discharge body weight values. We did not find differences between olanzapine and other antipsychotics regarding weight gain. In summary, we found that antipsychotics did not help with weight gain in youth with anorexia nervosa.

**Supplementary Information:**

The online version contains supplementary material available at 10.1186/s40337-023-00862-4.

## Background

Anorexia nervosa (AN) is a severe mental illness that affects mostly females [1–3], has typically an adolescent-onset [2, 4], and has one of the highest mortality rates among psychiatric diseases [5, 6]. In adolescents, an average prevalence of 0.3% has been reported [4]. Core symptoms of AN consist of self-induced underweight (due to food restriction, physical (hyper)activity, vomiting, use of laxatives or other methods), body image distortion up to delusional proportions, obsessive fear of being “fat” despite being underweight, and endocrine dysfunction (International Classification of Diseases, 10th revision (ICD-10)) [7]. There are two AN subtypes - the restricting type (AN-R) and the binge-purging type (AN-BP) - and an atypical subtype (AN-A) that does not meet all criteria. AN often has a chronic course [8], and somatic [8] as well as psychiatric comorbidities are very common (up to 70%), particularly depression, anxiety and obsessive–compulsive disorders [9, 10].

Being one of the most severe psychiatric disorders in youth, a multimodal treatment approach for AN is required, starting with medical stabilization and re-nutrition. Family-based therapy and, to a lesser extent, adolescent-focused psychotherapy, have proven efficacy in youth with AN [11–18]. However, limited illness insight and treatment motivation, which are associated with AN, challenge effective management. Given these illness features and psychiatric comorbidities, psychotropic medications are not infrequently used for patients with AN aiming to improve outcomes [19].

Over the years, several psychotropic drug groups, particularly, antidepressants, mood stabilizers, and antipsychotics, have been tested for the treatment of AN augmenting dietary and psychological treatments [20]. Currently, mainly antipsychotics are prescribed clinically, as antidepressants seem to be ineffective in AN, at least based on non-controlled, naturalistic data [20] in both adults [21] and adolescents [22, 23]. Reasons for antipsychotic use in AN are the almost delusional and ego-syntonic beliefs in AN (being too “fat”), the antidepressant and anxiolytic effects of (some) antipsychotics, their tranquilizing effect on agitation and physical hyperactivity [24] as well as the side effects of weight gain and sedation [25–28].

The first study with antipsychotic medications in 44 adult female inpatients with AN was conducted in 1958, comparing chlorpromazine plus insulin versus treatment as usual (TAU) [29]. Because of considerable side effects, the administration of chlorpromazine was quickly stopped.

In the 1980s, smaller, randomized controlled studies (RCTs) in women with AN were conducted with the first-generation antipsychotics (FGAs) pimozide and sulpiride, which did not produce any significant improvement in weight gain or disordered cognitions [30, 31]. To our knowledge, so far only two studies with the FGA haloperidol as a treatment option in adults with severe AN have been conducted. The six-month open trial of haloperidol in 13 adults with AN found significant changes in AN-related psychopathology, global illness severity, and body mass index (BMI) [32]. The nine-case chart review study in adults with severe AN-R with delusional body image disturbance reported a subjective reduction of 'delusional' body image disturbance with good tolerability of low-dose haloperidol [33].

The introduction of second-generation antipsychotics (SGAs) in the 1990s renewed the interest in antipsychotics for the treatment of AN, with subsequent RCTs (none exclusively in children and adolescents) and meta-analyses, which so far have not been able to demonstrate the superiority of SGAs versus placebo for weight gain and AN-related cognitive distortions [20, 26, 34–38]. However, one more recent, 16-week RCT in adult outpatients with AN (96% female), the largest to date (n = 152), did show significantly greater weight gain (0.165 kg/m^2^ per month) and greater improvements in shape concerns with olanzapine versus placebo, without significantly greater changes versus placebo in obsessions and metabolic parameters [39]. A very recent meta-analysis examined 7 studies regarding the effect of olanzapine on weight gain (304 patients with AN-R or AN-BP, 4 RCTs in adults (olanzapine vs. placebo), 3 studies in adolescents (age 12.3–21.8 years)). BMI at the end of treatment as primary outcome was significantly higher in adults (0.67 kg/m^2^, follow-up 8–16 weeks), whereas in adolescents, olanzapine as adjuvant treatment showed a non-significant increase in BMI (0.66 kg/m^2^, follow-up 10–26 weeks) due to the small sample size (65 participants in total) [40].

Currently, there are only a few small and only uncontrolled studies on the use of antipsychotics in AN in children and adolescents (from here on called “youth”) that provide heterogeneous results: Several case studies (total n = 14) showed promising results for olanzapine use on weight gain and eating disorder (ED)-psychopathology (in- and outpatients, age 10–18 years) [41–43]. Two open-label studies (total n = 45, mostly inpatients, age 8–17 years) showed significant weight gain [44, 45], but only the smaller trial (n = 13) also found significant improvement in ED-related psychopathology (global functioning, ED-related attitudes, anxious-depressive symptoms, and hyperactivity) [44].

The first retrospective chart review in youth with AN treated with olanzapine (n = 43, age 10–17 years, all levels of care) showed the following significant differences between olanzapine-treated youth and a matched sample of youth not receiving an antipsychotic: more comorbidities, lower admission BMI, longer treatment duration, higher number of treatments/admissions due to AN, and non-significantly higher psychopathology in the olanzapine group [46]. In the small subgroup with data on weight change (n = 11, only inpatients), those on olanzapine had non-significant differences in admission BMI, significantly more depressive symptoms, numerically higher weight gain per week, significantly higher discharge BMI (most likely due to the also longer treatment duration), and significantly more readmissions compared to the matched sample without antipsychotic medication [46]. In a second retrospective chart review, 22 of 106 adolescents (mean age = 14–15 years, inpatient or partial hospital program) received aripiprazole and were reported to have greater increases in BMI and BMI percentile than the remaining 84 adolescents not treated with an antipsychotic [47]. However, results were at least partially confounded by non-significantly longer treatment durations in the aripiprazole-treated group in the inpatients (p = 0.297) and, especially, day hospital patients (*p* = 0.061). Furthermore, another recent retrospective inpatient study of 79 youth (age: 14.6 ± 2.0 years, 98.7% female) examined the effect of the timing of SGA and nasogastric tube (NGT) initiation. Dividing patients into groups of early (0–7 days) or late (≥ 8 days) introduction of SGA and NGT, the authors found no difference in BMI change at discharge, but the length of stay was significantly shorter in the early/early group compared to late/late or late SGA/early NGT group, suggesting the potential utility of early SGA use [48].

Nevertheless, the first RCT conducted mostly in adolescents and young adult women with AN-R (double-blind, n = 20, 100% females, age 12–21 years, mean age = 17.1, all levels of care) showed neither significant weight change nor improvement in general psychopathology assessments or eating attitudes and behavior with olanzapine versus placebo [49]. Additionally, the first RCT of risperidone versus placebo (double-blind, n = 40, 100% females, age 12–21 years, mean age = 16, inpatient and day treatment) also did not find significant differences for weight change or drive for thinness, body dissatisfaction, and body image distortion [50].

Based on the limited randomized data, international and national guidelines do not consider the evidence strong enough to recommend antipsychotic treatment for the management of AN, both for weight restoration and for ED psychopathology [14–16, 51].

However, despite the mostly negative meta-analytic evidence for benefits in adults with AN [20, 26, 34–38] and virtually absent data in youth, psychotropic drugs are often prescribed in AN in real-world settings [19, 52]. Naturalistic data capture patients who may not be included in RCTs that have strict in- and exclusion criteria. Therefore, information from clinical practice settings can be complementary and informative for clinical care and future RCTs. For example, a multicenter retrospective study in youth and a few young adults (n = 635 at baseline, n = 359 with 1-year follow-up, mean age = 15.3 years) with restrictive eating disorders (AN, AN-A, avoidant/restrictive food intake disorder (ARFID)), a high rate of comorbidities and different levels of care, reported psychotropic drug use in 21.5% of the youth at the start of treatment and 57% after one year, of which 82.5% were selective serotonin reuptake inhibitors (SSRIs) and 16.8% (n = 60) antipsychotics. As many as 30.1% of the youth on psychotropic drugs took two or more medications. However, in this naturalistic setting, there was no significant increase in weight restoration compared to patients without medication [52].

To our knowledge, that retrospective chart review [52] is the largest naturalistic study assessing the efficacy of antipsychotic treatment of inpatient youth with AN. Based on the relatively frequent use of antipsychotics in youth with AN and limited information on the potential benefits [53], the current retrospective chart review study aimed to assess i) the clinical correlates of antipsychotic use in youth with AN, ii) specific antipsychotic choice during inpatient treatment, and iii) effects of (specific) antipsychotics regarding inpatient weight gain in comparison to unmatched and to carefully matched non-antipsychotic treated youth. Additionally, we sought to compare (specific) antipsychotic medication treatment periods versus non-antipsychotic treated time periods in the same patient in order to evaluate within-subject alterations of weight gain trajectories off versus on antipsychotic treatment. We hypothesized that antipsychotic use would be associated with greater psychiatric comorbidity, lower baseline body composition markers as well as less initial improvement in body composition parameters compared to non-antipsychotic-treated youth, but with significantly greater increases in body weight after antipsychotic initiation in within-subject analyses.

## Methods

This was a retrospective cohort study conducted at the tertiary care university inpatient department of the Charité-Universitaetsmedizin Berlin, Germany. The primary study aim was to compare weight trajectories and other correlates in youth with AN treated with versus without antipsychotics.

### Inclusion criteria and setting

Patient records were searched manually for all youth ages 8–18 years who were consecutively admitted for the first time to the university inpatient department between 1992 and 2015 with an ICD-10 diagnosis of AN, restricting type (AN-R, F50.00) or binge-purge type (AN-BP, F50.01). All psychiatric diagnoses were made clinically based on ICD-10 criteria [7] at hospital admission and coded in a digital data extraction form.

All patients received a German guideline-consistent multimodal eating disorder-specific cognitive-behavioral therapy-based program, including supervised meals, nutritional counseling, individual cognitive behavioral or psychodynamically-informed therapy and group psychotherapy, family psychoeducation, body therapy, and dialectic-behavioral therapy-skills training, attending also the hospital's school.

### Exclusion criteria

We excluded patients with a length of stay < 2 weeks, lack of specified AN subtype, AN not meeting ICD-10 criteria, lack of BMI percentile data at admission and discharge, and lack of information about treatment duration.

### Data extraction

Data were retrieved manually by independent coders from patients' records drawing on a standardized departmental admission variable data set, which was supplemented by hand-searched information from admission, intra-treatment, and discharge documentation to gather detailed data on changes in weight and height, psychotropic medication treatment, and reasons for a change in antipsychotic treatment during inpatient care. The data abstractors were trained before data collection, supervised and monitored by CJ, and applied a standardized and study-specific template using the statistical package for social sciences (SPSS) software [54]. The data abstractors were blind to the study aims, hypotheses, and the compared study groups.

Collected data included patient demographics (sex, age at admission), anthropometric characteristics (BMI, BMI percentile, BMI z-scores, kg < first BMI percentile both at admission and discharge; weight gain in kg per week, BMI per week, BMI percentile per week, BMI z-scores per week), menstruation (age at menarche, primary vs. secondary amenorrhea, oligomenorrhea), illness/other clinical variables (age at onset of AN, duration of AN, AN subtype, other psychiatric diagnoses, intelligence level, history of childhood abuse (physical, emotional, sexual), family psychopathology), and treatment variables (treatment duration, antipsychotic treatment (i.e., type(s), maximum and end dose(s), time(s) of start and stop, reason(s) for stopping), type of other psychotropic medication treatment during hospitalization).

### Ethics committee approval

The Ethics Committee of the Charité-Universitaetsmedizin Berlin approved the study; individual patient's/guardian's consent was not required, as this was a retrospective cohort study.

### Statistical analysis

The matched sample subgroup analyses were preplanned sensitivity analyses to overcome any significant differences in the compared study groups that received clinically chosen treatment.

We compared patients with versus without antipsychotic treatment, using independent* t*-tests for continuous variables with normal distribution and Mann-Whitney U-test for continuous variables with non-normal distribution, as well as χ^2^-tests (with continuity correction when cell size was 5–9) or Fisher's exact test (when cell size was < 5) for categorial variables. A sample size calculation was not performed, as all inpatients with AN consecutively admitted between 1992 and 2015 were included in the study.

To analyze the effect-size of group differences, we used the following calculations based on the nature of the data: for Chi-Square-test: Cramer's V (small effect: 0.1, medium effect: 0.3, large effect: 0.5, range of values: 0 to +1), for Mann-Whitney U-test: r = (z/√N) (small effect: 0.1, medium effect: 0.3, large effect: 0.5, range of values: 0 to + 1), for Wilcoxon test: r = (z/√N) (small effect: 0.1, medium effect: 0.3, large effect: 0.5, range of values: 0 to + 1), and for t-test: Cohen's d: r = (mn1 − mn2/SD) (small effect: 0.2, medium effect: 0.5, large effect: 0.8, range of values: 0 to + 1) [55].

To determine potential differences in the effect by antipsychotic type, we compared anthropometric parameters in patients treated with olanzapine (the largest group) versus non-olanzapine antipsychotics, using Mann-Whitney U-test.

To determine independent factors associated with antipsychotic use, we performed a stepwise backward elimination multivariable logistic regression comparing patients with versus without antipsychotics and entering all variables with *p* < 0.05 in the univariate analysis into the initial model.

Furthermore, youth with antipsychotic treatment were compared to a subset of youth without antipsychotic treatment matched on 14 clinical variables that were either set (sex, year of hospital admission) or significantly different between youth with versus without antipsychotics in univariate analyses (age of ED onset, ED subtype, duration of illness, age at hospital admission, history of childhood emotional abuse, history of childhood physical abuse, at least one psychiatric comorbidity, average number of psychiatric comorbidities, major depressive disorder (MDD), borderline personality disorder traits, at least one antidepressant, treatment duration). In the matched subsample 1, all patients were required to have admission and discharge anthropometric data. In the matched subsample 2, which was used to compare weight trajectories before versus after antipsychotic use, patients on antipsychotics were required to also have information on height and weight at the time of antipsychotic onset and offset. The control group of the matched subsample 2 was required to have height and weight data at 5 weeks post admission (median time of the initiation of antipsychotic treatment in the antipsychotic-treated group). Within and across group comparisons of the weight trajectories were performed using Wilcoxon-test and Mann-Whitney U-test, respectively.

Furthermore, using the median split on weight change/week, we sought to identify correlates of faster versus slower weight gain, in order to see if antipsychotic use would be a predictor of faster weight gain overall. Finally, a descriptive visual plot was created for each individual patient, coding the weight change before, during, and after antipsychotic treatment (as applicable).

All analyses were conducted in the SPSS software [54] and with α = 0.05.

## Results

### Population and treatment

The initial sample consisted of 331 female and male youth of whom 37 were excluded because the AN subtype was not specified (n = 7), > 10th BMI percentile at hospital admission (n = 18), no BMI percentile information at hospital admission (n = 2), AN-R or AN-BP with regular menstruation (n = 3), treatment duration < 14 days (n = 3), no information about treatment duration (n = 4).

Altogether, 294 youth with AN-R (238, 81.0%) or AN-BP (56, 19.0%) were included in the analyses (Table 1). Data for the primary focus of this study, i.e., anthropometric changes from baseline to endpoint, were complete, except for missing data in one patient (0.3%) regarding discharge BMI, discharge BMI percentile, weekly change in weight, BMI and BMI percentile, or for 2 patients (0.7%) regarding discharge BMI z-score and weekly change in BMI z-score.

**Table 1:** Clinical characteristics of anorexia nervosa patients with versus without antipsychotic medication

	Total (n = 294)	With antipsychotics (n = 44)	Without antipsychotics (n = 250)	t-test Mann–Whitney U-test χ^2^-test			
	M ± SD Mdn (Q1, Q3) n (%)	M ± SD Mdn (Q1, Q3) n (%)	M ± SD Mdn (Q1, Q3) n (%)	df z	t U χ^2^	p	Cohens d Cramers V
ED diagnoses	–	–	–	1	2.3	0.132	0.1
AN-R	238 (81.0)	32 (72.7)	206 (82.4)	–	–	–	–
AN-BP	56 (19.0)	12 (27.3)	44 (17.6)	–	–	–	–
Sex	–	–	–	1	0.0	1.000	0.0
Female	284 (96.6)	43 (97.7)	241 (96.4)	–	–	–	–
Male	10 (3.4)	1 (2.3)	9 (3.6)	–	–	–	–
Age of onset, years	14.1 (13.0, 15.2)	13.6 (12.2, 14.4)	14.2 (13.1, 15.3)	− 2.9	3948.0	0.003*	0.2
Duration of illness, months	12.6 ± 12.1	13.9 ± 12.5	12.4 ± 12.0	290	− 0.8	0.449	0.1
Age at admission, years	15.2 (14.0, 16.6)	14.8 (13.2, 15.9)	15.4 (14.1, 16.6)	− 2.5	4204.0	0.013*	0.2
Menarche, age in years	12.0 ± 2.0	12.0 ± 2.0	12.0 ± 1.9	− 0.6	1238.0	0.574	0.0
Primary amenorrhea	55 (18.7)	14 (31.8)	41 (16.4)	1	5.8	0.016*	0.1
Secondary amenorrhea	184 (62.6)	23 (52.3)	161 (64.4)	1	2.4	0.125	0.1
Oligomenorrhea	9 (3.1)	1 (2.3)	8 (3.2)	1	0.0	1.000	0.0
Intelligence							
Very high intelligence	15 (5.1)	2 (4.5)	13 (5.2)	1	0.0	1.000	0.0
High intelligence	99 (33.7)	20 (45.5)	79 (31.6)	1	3.2	0.073	0.1
Average intelligence	176 (59.9)	21 (47.7)	155 (62.0)	1	3.2	0.075	0.1
Below average intelligence	4 (1.4)	1 (2.3)	3 (1.2)	1	0.0	0.479	0.0
Family psychopathology and childhood abuse							
Family psychopathology present	194 (66.0)	32 (72.7)	162 (64.8)	1	1.0	0.389	0.1
History of childhood abuse	26 (8.8)	9 (20.5)	17 (6.8)	1	7.0	0.008*	0.2
Emotional	9 (3.1)	5 (11.4)	4 (1.6)	1	9.0	0.001*	0.2
Physical	12 (4.1)	5 (11.4)	7 (2.8)	1	5.0	0.025*	0.2
Sexual	7 (2.4)	2 (4.6)	5 (2.0)	1	0.2	0.282	0.1
Psychiatric comorbidities							
Comorbidities, average number	0.0 (0.0, 0.1)	1.0 (0.0, 1.0)	0.0 (0.0, 1.0)	− 3.3	3973.0	0.001*	0.2
At least one comorbidity	129 (43.9)	29 (65.9)	100 (40.0)	1	10.2	0.001*	0.2
Substance use disorder	3 (1.0)	1 (2.3)	2 (0.8)	1	0.0	0.386	0.1
Affective disorders	82 (27.9)	21 (47.7)	61 (24.4)	1	10.12	0.001*	0.2
Major depressive disorder	48 (16.3)	14 (31.8)	34 (13.6)	1	9.1	0.003*	0.2
Persistent affective disorder	39 (13.3)	8 (18.2)	31 (12.4)	1	0.6	0.423	0.1
Neurotic, stress, and somatoform disorders	44 (15.0)	8 (18.2)	36 (14.4)	1	0.2	0.675	0.0
Phobia	7 (2.4)	1 (2.3)	6 (2.4)	1	0.0	1.000	0.0
Obsessive–compulsive disorder	28 (9.5)	6 (13.6)	22 (8.8)	1	0.5	0.466	0.1
Post-traumatic stress disorder	2 (0.7)	0 (0.0)	2 (0.8)	1	0.0	1.000	0.0
Personality and behavioral disorder traits	22 (7.5)	7 (15.9)	15 (6.0)	1	4.0	0.046*	0.1
Borderline	9 (3.1)	4 (9.1)	5 (2.0)	1	4.2	0.041*	0.1
Compulsive	4 (1.4)	1 (2.3)	3 (1.2)	1	0.0	0.479	0.0
Disorders with onset in childhood/adolescence	5 (1.7)	0 (0)	5 (2.0)	1	0.1	1.000	0.1
Anthropometric Characteristics at Baseline							
BMI at admission	14.4 (13.6, 15.3)	14.4 (13.4, 15.0)	14.4 (13.6, 15.4)	− 1.2	4888.5	0.239	0.1
BMI percentile at admission	1.0 (1.0, 1.0)	1.0 (1.0, 1.0)	1.0 (1.0, 1.0)	− 0.2	5442.5	0.872	0.0
BMI z-scores at admission	− 3.0 (− 3.8, − 2.3)	− 2.8 (− 3.7, − 2.4)	− 3.0 (− 3.8, − 2.3)	− 0.4	5300.0	0.701	0.0
Kg < 1. BMI percentile at admission	− 1.8 (− 4.1, 0.0)	− 1.8 (− 3.6, 0.0)	− 1.8 (− 4.3, 0.0)	− 0.1	5467.0	0.948	0.0
Anthropometric Characteristics at Discharge							
BMI at discharge	17.4 (16.6, 18.0)	16.9 (16.0, 17.5)	17.5 (16.7, 18.0)	− 2.8	3942.5	0.005*	0.2
BMI percentile at discharge	10.0 (5.5, 16.3)	9.0 (3, 14)	10.0 (6.0, 17.0)	− 1.3	4722.0	0.203	0.1
BMI z-scores at discharge	− 1.3 (− 1.6, − 1.0)	− 1.4 (− 2.0, − 1.1)	− 1.3 (− 1.5, − 1.0)	− 1.6	4520.5	0.103	0.1
Weight change, kg/week	0.5 (0.4, 0.7)	0.4 (0.3, 0.6)	0.5 (0.4, 0.7)	− 4.3	3148.5	< 0.001*	0.3
BMI change/week	0.2 (0.1, 0.3)	0.1 (0.1, 0.2)	0.2 (0.1, 0.3)	− 3.4	3653.0	0.001*	0.2
BMI percentiles change/week	0.7 (0.3, 1.1)	0.3 (0.1, 0.9)	0.7 (0.3, 1.2)	− 2.8	3947.0	0.005*	0.2
BMI z-score change/week	0.1 (0.1, 0.2)	0.1 (0.1, 0.1)	0.1 (0.1,0.2)	− 3.6	3515.0	< 0.001*	0.2
Treatment duration, days	98.2 ± 43.2	124.1 ± 55.4	93.7 ± 39.2	50.8	− 3.5	< 0.001*	0.6
Psychotropic medication treatment other than antipsychotics during hospitalization							
Psychotropic medication other than antipsychotics	56 (19)	20 (46)	36 (14)	1	23.4	< 0.001*	0.3
One	50 (17)	15 (34)	35 (14)	–	–	–	–
Two	4 (1)	3 (7)	1 (4)	–	–	–	–
Three	0 (0)	2 (5)	0 (0)	–	–	–	–
Four	2 (7)	0 (0)	0 (0)	–	–	–	–
Antidepressants, average number	0.0 (0.0, 1.0)	0.0 (1.0, 2.0)	0.0 (0.0, 0.0)	− 5.0	3738.0	< 0.001*	0.3
At least one antidepressant	56 (19.0)	20 (45.5)	36 (14.4)	1	23.4	< 0.001*	0.3
SSNRI	2 (0.7)	1 (2.3)	1 (0.4)	1	1.9	0.277	0.1
Venlafaxine	2 (0.7)	1 (2.3)	1 (0.4)	1	1.9	0.277	0.1
SSRI, at least one	47 (16.0)	16 (36.4)	31 (12.4)	1	16.0	< 0.001*	0.3
Escitalopram	22 (7.5)	8 (18.2)	14 (5.6)	1	8.6	0.003*	0.2
Fluoxetine	14 (4.8)	5 (11.4)	9 (3.6)	1	3.4	0.065	0.1
Fluvoxamine	7 (2.4)	2 (4.5)	5 (2.0)	1	0.2	0.628	0.1
Paroxetine	2 (0.7)	1 (2.3)	1 (0.4)	1	0.2	0.277	0.1
Tetracyclic antidepressant	6 (2.0)	5 (11.4)	1 (0.4)	1	17.3	< 0.001*	0.3
Mirtazapine	6 (2.0)	5 (11.4)	1 (0.4)	1	17.3	< 0.001*	0.3
Tricyclic antidepressant	2 (0.7)	1 (2.3)	1 (0.4)	1	0.2	0.277	0.1
Doxepin	2 (0.7)	1 (2.3)	1 (0.4)	1	0.2	0.277	0.1
Anxiolytic	2 (0.7)	2 (4.5)	0 (0.0)	1	5.7	0.022*	0.2
Lorazepam	2 (0.7)	2 (4.5)	0 (0.0)	1	5.7	0.022*	0.2

*Note*. AN-BP = anorexia nervosa, binge-purge type, AN-R = AN, restricting type, BN = bulimia nervosa, intelligence = very high (IQ > 129), high (IQ 115–129), average (IQ 85–114), below average (IQ 70–84), IQR = interquartile range, Mdn = median, SD = standard deviation, * p < *0.05*

At the time of admission, the 294 youth had a median age (interquartile range) of 15.2 (14.0, 16.6) years, 96.6% were female, the mean AN illness duration was 12.6 ± 12.1 months, 18.7% had primary amenorrhea and 62.6% had secondary amenorrhea. Of the 294 included youth with AN, 44 patients (15.0%) were prescribed antipsychotics and 250 (85.0%) were not. The 44 patients received altogether 52 antipsychotic trials, resulting in a mean number of 1.2 ± 0.6 antipsychotics per patient on antipsychotics during the inpatient stay (Table 2).

**Table 2:** Prevalence and characteristics of antipsychotic medication in AN

	Total (n = 44)
Prevalence of antipsychotics	M ± SD N (%)
Antipsychotics, average number	1.2 ± 0.6
One antipsychotic	37 (84.1)
Two antipsychotics	6 (13.6)
Three antipsychotics	0 (0)
Four antipsychotics	1 (2.3)
Total number of antipsychotic trials	52 (100)
Typical antipsychotics, number	13 (25.0)
Chlorprothixene, number	11 (21.2)
Pipamperone, number	1 (1.9)
Thioridazine, number	1 (1.9)
Atypical antipsychotics, number	39 (75.0)
Olanzapine, number	33 (63.5)
Quetiapine, number	5 (9.6)
Risperidone, number	1 (1.9)
Characteristics of antipsychotics	M ± SD N (%)
Start week of antipsychotics, number	5.9 ± 3.8
End week of antipsychotics, number	12.7 ± 5.5
Duration of antipsychotics, average weeks	6.8 ± 4.0
Duration of antipsychotics, percentage of treatment duration, average weeks	43.0 ± 25.0
Duration of treatment weeks after antipsychotics offset	6.0 ± 6.9
Maximum dose of antipsychotics, mg (CPZ equivalents)	34.5 ± 75.2
End dose of antipsychotics, mg (CPZ equivalents)	34.7 ± 89.7
Antipsychotics after discharge	26 (59.1)
Changing/ending antipsychotic medication	M ± SD N (%)
Reasons for changing/ending antipsychotics	15 (34.1)
Inefficiency	6 (13.6)
Intolerability	4 (9.1)
No longer needed	4 (9.1)
Patient or parent request	1 (2.3)
Other	0 (0)

**Fig. 1:**
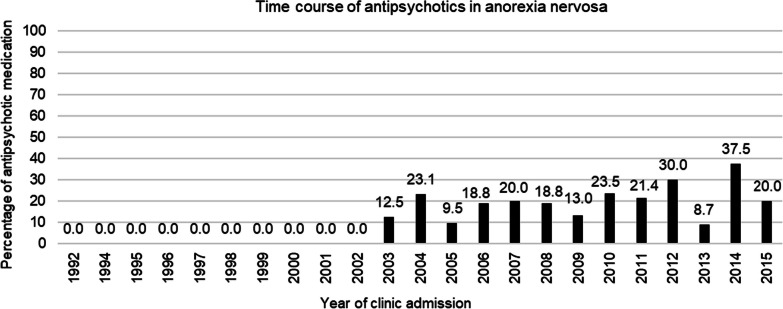
Time course of antipsychotic medication in anorexia nervosa

Six different antipsychotics were prescribed, with olanzapine (n = 33, 63.5%) being by far the most frequently used antipsychotic among the 39 SGA (75.0%) and 13 FGA (25.0%) trials. The antipsychotic prescription frequency increased significantly during the observation period from 1992 to 2015 (*p* = 0.001) and even during the observation period from 1999 to 2015 (*p* = 0.042) (Fig. 1).

Antipsychotics were started at the mean inpatient treatment time of week 5.9 ± 3.8 and ended at 12.7 ± 5.5 weeks, resulting in an average duration of 6.8 ± 4.0 weeks. The average treatment duration after the antipsychotics offset was 6.0 ± 6.9 weeks, with 26 patients (59.1%) still taking antipsychotics at the time of discharge. In 15 cases (34.1%) antipsychotic medication was changed or stopped, with reasons for this being heterogenous (Table 2).

### Baseline correlates of antipsychotic use

Youth treated with versus without antipsychotics differed significantly on the following baseline variables: younger age at AN onset and at admission, more often primary amenorrhea, longer duration of illness, more often history of childhood abuse (i.e., total, emotional, and physical abuse), and higher number of psychiatric comorbidities (i.e., MDD, borderline personality disorder traits) (Table 1).

In a binary logistic regression analysis, antipsychotic medication use was independently associated with younger age of AN illness onset, history of emotional childhood abuse, and at least one psychiatric comorbidity at baseline (*p* < 0.0001) (Table 3).

**Table 3:** Binary logistic regression model: Baseline predictors (moderators), and baseline predictors (moderators) together with intra-treatment predictors (mediators) of clinical antipsychotic medication use during inpatient treatment of youth with anorexia nervosa

Nagelkerke r^2^	Variables	B	S.E	Wald	df	p	Exp (B)	95% CI	
								Lower	Upper
Baseline predictors (moderators) of inpatient antipsychotic treatment in youth with anorexia nervosa									
Overall Model: 0.160 *p* < 0.0001	Age of onset	− 0.3	0.1	9.6	1	0.002*	0.7	0.6	0.9
	Emotional childhood abuse	− 1.9	0.8	6.3	1	0.012*	0.2	0.0	0.7
	At least one comorbidity	0.7	0.2	10.7	1	0.001*	2.0	1.3	3.0
Baseline (moderators) and intra-treatment (mediators) predictors of inpatient antipsychotic treatment in youth with anorexia nervosa									
Overall Model: 0.373 *p* < 0.0001	Age at admission	− 0.4	0.1	6.6	1	0.010*	0.7	0.5	0.9
	History of physical childhood abuse	− 2.2	0.9	5.5	1	0.019*	0.1	0.0	0.7
	Borderline personality disorder traits	− 2.6	1.0	6.5	1	0.011*	0.1	0.0	0.5
	Weight change before antipsychotics administration, kg/week	− 3.6	1.0	11.5	1	0.001*	0.0	0.0	0.2

Note. * p < *0.05*

### Outcomes associated with antipsychotic treatment

In the total sample, BMI at discharge and all weight change parameters showed lower weight gain trajectories for patients treated with antipsychotics, while anthropometric characteristics at admission had not differed significantly (Table 1). Furthermore, youth treated with versus without antipsychotics received significantly more often other psychotropic medication (mostly antidepressants) during hospitalization (Table 1).

In a multivariable logistic regression analysis (Table 3), antipsychotic medication use was independently associated with younger age at admission, history of physical childhood abuse, comorbid borderline personality disorder traits, and less weight change before antipsychotic administration (kg/week) (*p* < 0.0001).

To rule out a selection by indication bias, we matched the antipsychotic-treated group (n = 43, one patient was dropped because there was no weight information at discharge) with a subsample of 43 youth without antipsychotics, matching on the 14 clinical variables that included AN subtype and sex, as well as all 12 variables that were significantly associated with antipsychotic use versus no use (Table 4). In this matched sample 1, anthropometric characteristics at admission were not significantly different while all anthropometric parameters at discharge as well as BMI percentiles change per week were significantly lower in the antipsychotic-treated group.

**Table 4:** Clinical characteristics of anorexia nervosa patients with versus without antipsychotic medication—matched sample 1

	Total (n = 86)	With antipsychotics (n = 43)	Without Antipsychotics (n = 43)	t-test Mann–Whitney U-test χ^2^-test			Cohens d Cramers V
	M ± SD Mdn (Q1, Q3) n (%)	M ± SD Mdn (Q1, Q3) n (%)	M ± SD Mdn (Q1, Q3) n (%)	df z	t U χ^2^	p	
ED diagnoses	–	–	–	1	0.0	1.000	0.0
AN-R	64 (74)	32 (74)	32 (74)	–	–	–	
AN-BP	22 (26)	11 (26)	11 (26)	–	–	–	
Sex	–	–	–	1	0.0	1.000	0.0
Female	84 (98)	42 (98)	42 (98)	–	–	–	
Male	2 (2)	1 (2)	1 (2)	–	–	–	
Age of onset, years	13.6 ± 2.1	13.6 ± 2.2	13.5 ± 2.3	− 0.2	904.0	0.859	0.2
Duration of illness, months	13.4 ± 1.4	13.2 ± 11.8	13.5 ± 13.8	84	0.1	0.913	0.1
Age at admission, years	14.6 ± 2.6	14.9 ± 2.9	14.5 ± 2.7	− 0.2	899.0	0.826	0.1
History of childhood emotional abuse	9 (11)	5 (12)	4 (9)	1	0.1	1.000	0.0
History of childhood physical abuse	8 (9)	5 (12)	3 (7)	1	0.6	0.713	0.1
Comorbidities, average number	1.0 (0.0, 1.0)	1.0 (0.0, 1.0)	1.0 (0.0, 1.0)	− 0.4	881.5	0.657	0.1
At least one comorbidity	55 (64)	28 (65)	27 (63)	1	0.1	0.822	0.0
Major depressive disorder	29 (34)	14 (33)	15 (35)	1	0.5	0.820	0.0
Borderline personality disorder traits	6 (7)	3 (7)	3 (7)	1	0.0	1.000	0.0
Anthropometric characteristics at baseline							
BMI at admission	14.3 ± 1.7	14.4 ± 1.7	14.2 ± 2.1	− 0.4	881.0	0.707	0.0
BMI percentile at admission	1.0 ± 0.0	1.0 ± 0.0	1.0 ± 2.0	− 1.1	829.5	0.267	0.0
BMI z-scores at admission	− 2.9 ± 1.5	− 2.9 ± 1.4	− 2.9 ± 1.8	− 0.4	882.0	0.714	0.1
Kg < 1. BMI percentile at admission	− 1.6 ± 3.7	− 1.6 ± 3.6	− 1.5 ± 4.4	− 0.1	909.5	0.894	0.1
Anthropometric characteristics at discharge							
BMI at discharge	17.2 ± 1.5	17.0 ± 1.5	17.4 ± 1.2	− 2.5	632.5	0.012*	0.6
BMI percentile at discharge	12.0 ± 13.0	9.0 ± 11.0	14.0 ± 10.0	− 2.5	639.0	0.014*	0.4
BMI z-scores at discharge	− 1.2 ± 0.7	− 1.4 ± 0.9	− 1.0 ± 0.5	− 2.8	601.0	0.005*	0.5
Weight change, kg/week	0.4 ± 0.3	0.4 ± 0.4	0.4 ± 0.3	− 1.9	710.0	0.064	0.5
BMI change/week	0.2 ± 0.1	0.1 ± 0.1	0.2 ± 0.1	− 1.3	773.5	0.192	0.3
BMI percentiles change/week	0.6 ± 0.8	0.3 ± 0.8	0.8 ± 0.7	− 2.6	624.5	0.010*	0.5
BMI z-score change/week	0.1 ± 0.1	0.1 ± 0.1	0.1 ± 0.1	− 1.4	769.0	0.179	0.4
Treatment duration, days	118.1 ± 4.8	122.1 ± 54.5	114.1 ± 30.5	84	− 0.8	0.400	0.0
Psychotropic medication other than antipsychotics during hospitalization							
Psychotropic medication other than antipsychotics	33 (38.4)	19 (44.2)	14 (32.6)	1	1.2	0.268	0.1
One	28 (32.6)	14 (32.6)	14 (32.6)	–	–	–	–
Two	3 (3.5)	3 (7.0)	0 (0.0)	–	–	–	–
Three	2 (2.3)	2 (4.7)	0 (0.0)	–	–	–	–
Four	0 (0.0)	0 (0.0)	0 (0.0)	–	–	–	–
Antidepressants, average number	0.0 (0.0,1.0)	0.0 (0.0,1.0)	0.0 (0.0,1.0)	− 1.4	789.00	0.170	0.3
At least one antidepressant	33 (38.4)	19 (44.2)	14 (32.6)	1	1.2	0.268	0.1
SSNRI	2 (2.3)	1 (2.3)	1 (2.3)	1	0.0	1.000	0.0
Venlafaxine	2 (2.3)	1 (2.3)	1 (2.3)	1	0.0	1.000	0.0
SSRI, at least one	28 (32.6)	16 (37.2)	12 (27.9)	1	0.8	0.357	0.1
Escitalopram	14 (16.3)	8 (18.6)	6 (14.0)	1	0.3	0.559	0.1
Fluoxetine	9 (10.5)	5 (11.6)	4 (9.3)	1	0.1	0.724	0.0
Fluvoxamine	4 (4.7)	2 (4.7)	2 (4.7)	1	0.0	1.000	0.0
Paroxetine	1 (1.2)	1 (2.3)	0 (0.0)	1	0.0	0.237	0.1
Tetracyclic antidepressant	5 (5.8)	5 (11.6)	0 (0.0)	1	7.2	0.055	0.2
Mirtazapine	5 (5.8)	5 (11.6)	0 (0.0)	1	7.2	0.055	0.2
Tricyclic antidepressant	1 (1.2)	1 (2.3)	0 (0.0)	1	1.4	1.000	0.1
Doxepin	1 (1.2)	1 (2.3)	0 (0.0)	1	1.4	1.000	0.1
Anxiolytic	2 (2.3)	2 (4.7)	0 (0.0)	1	2.8	0.494	0.2
Lorazepam	2 (2.3)	2 (4.7)	0 (0.0)	1	2.8	0.494	0.2

*Note*. AN-BP = anorexia nervosa, binge-purge type, AN-R = AN, restricting type, BN = bulimia nervosa, IQR = interquartile range, Mdn = median, SD = standard deviation, * p < *0.05*

### Patients prescribed antipsychotics

Comparing youth treated with olanzapine versus those treated with other antipsychotics to exclude a bias due to less weight-gain producing, non-olanzapine antipsychotics, all anthropometric characteristics at admission were significantly lower in the olanzapine group, except for admission BMI (Additional file 1: Table S1). However, all weight changes and anthropometric characteristics at discharge did not differ significantly, with a trend towards lower values in the olanzapine group.

Furthermore, comparing youth who received antipsychotics until the time of discharge with those who stopped before discharge regarding anthropometric characteristics, there were no significant differences in anthropometric outcomes (Additional file 1: Table S2).

To compare weight change before and after antipsychotics in the same patient and compare that to similar times during hospitalization in the non-antipsychotic group, we created an artificial split at treatment week 5 based on the median time point of antipsychotic initiation in the antipsychotic-treated group. Because data on weight change before and after the median split were not available for all patients, the antipsychotic-treated group size was reduced from n = 44 to n = 31, to which 31 non-antipsychotic treated youth were matched. For details of the demographic, illness and treatment characteristics of the matched sample 2, please see Additional file 1: Table S3. Similarly, the non-antipsychotic-treated group was reduced from n = 250 to n = 176 (Table 5). The weight change (kg/week) did not differ significantly within the antipsychotic and the non-antipsychotic group when comparing the anthropometric values before and after the artificial split (Table 5). Similarly, even when comparing the subsamples matched on the same 14 variables as above (n = 31 vs. n = 31) with available data (Additional file 1: Table S3), there were no significant differences in anthropometric changes before and after the median split of 5 weeks within both groups (Table 5).

**Table 5:** Weight change per week in kg before versus after antipsychotics: within-group comparison and between-group comparison

	Weight change before antipsychotic administration, kg/week	Weight change after antipsychotic administration, kg/week	Wilcoxon-test		n^2^
	Mdn (Q1,Q3)	Mdn (Q1,Q3)	W	p	
Weight change per week in kg before versus after antipsychotics: within-group comparison					
With antipsychotics (n = 31)	0.3 (0.2, 0.4)	0.3 (0.1, 0.4)	− 0.6	0.531	12.2
Without antipsychotics (n = 176)	0.4 (0.3, 0.6)	0.5 (0.3, 0.6)	− 0.3	0.761	12.0
Matched sample with antipsychotics (n = 31)	0.3 (0.2, 0.4)	0.3 (0.1, 0.4)	− 0.6	0.531	12.1
Matched sample without antipsychotics (n = 31)	0.4 (0.3, 0.5)	0.4 (0.3, 0.5)	− 0.8	0.433	12.1

	With antipsychotics (n = 31)	Without antipsychotics (n = 176)	Mann–Whitney U-test			Cohens d	Matched sample		Mann–Whitney U-test			Cohens d
							With antipsychotics (n = 31)	Without antipsychotics (n = 31)				
	Mdn (Q1,Q3)	Mdn (Q1,Q3)	Z	U	p		Mdn (Q1,Q3)	Mdn (Q1,Q3)	Z	U	p	
Weight change per week in kg before versus after antipsychotics: between-group comparison												
Weight change before antipsychotics administration, kg/week*	0.3 (0.2,0.4)	0.4 (0.3,0.6)	− 3.8	1552.0	< 0.001*	0.5	0.3 (0.2,0.4)	0.4 (0.3,0.5)	− 2.0	343.0	0.053	0.5
Weight change after antipsychotics administration, kg/week*	0.3 (0.1,0.4)	0.5 (0.3,0.6)	− 3.6	1623.0	< 0.001*	0.5	0.3 (0.1,0.4)	0.4(0.3,0.5)	− 2.7	287.0	0.006*	0.7

*Note.* IQR = interquartile range, Mdn = median, artificial median split at treatment week 5 for the group without antipsychotics, * p < 0.05

However, comparing the antipsychotic and the non-antipsychotic groups, youth on antipsychotics gained significantly less weight than patients without antipsychotics, both before and, especially, after the artificial median split at week 5 (Table 5). The same was evident in the matched sample (except for an only trend-significant lower weight gain for the time before the antipsychotic use in the antipsychotic-treated group (*p *= 0.053)).

Dividing youth receiving antipsychotics into those with slower (n = 22) versus faster (n = 21) weight gain on the median split at 0.354 kg/week, antipsychotic use was not significantly associated with either faster or slower weight gain, while faster weight gain was associated with less family psychopathology (52% vs. 91%) and less use of mirtazapine (0% vs. 22.7%), as well as, expectedly due to the group definition, higher discharge BMI and BMI percentile and shorter hospital stay (Additional file 1: Table S4).

Finally, within-patient analyses of weight change on versus off antipsychotic use (Additional file 1: Fig. S1) confirmed that no significant differences in weight gain trends were visible across on versus off antipsychotic time periods.

## Discussion and conclusions

The main findings of this retrospective cohort study of 294 consecutively hospitalized youth with AN followed for a mean of 3.2 months are that (1) altogether 15% of youth hospitalized for AN were prescribed antipsychotics, predominantly consisting of olanzapine (64%); (2) antipsychotic use in youth with AN increased significantly over time; (3) expectedly, antipsychotics were prescribed in more severely ill youth with AN and those with less initial weight gain; and (4) contrary to our hypotheses, clinician's choice of antipsychotic treatment did not significantly improve weight gain in youth with AN; rather weight gain trajectories per week remained below youth not prescribed antipsychotics. This negative finding was apparent in the total, unmatched sample as well as in a subsample carefully matched on 14 parameters that differed at baseline between youth with AN receiving versus not receiving antipsychotics. The negative result was also found when comparing weight change within antipsychotic-treated youth in a time trajectory analysis, comparing the change in weight/week before versus after antipsychotic use, both within antipsychotic-treated youth and between matched antipsychotic versus non-antipsychotic treated groups, using the median 5-week time point when antipsychotics were started as the pre-post mirror time point of the matched non-antipsychotic-treated sample. Furthermore, comparing patients with faster or slower weight gain, dividing groups by the median split of weight change/week across the inpatient treatment duration, antipsychotic use was unrelated to the speed of weight gain.

The finding that 15% of the total sample received antipsychotics is in the mid-range of frequencies of 8–22% reported in other retrospective chart studies of youth with AN ± other eating disorders in inpatient and outpatient settings, regardless of the type of antipsychotic used [19, 46, 47, 52, 56]. In our inpatient setting, antipsychotic use increased significantly from 1992 (0.0%) to 2015 (20.0%), and even from 1999 (10.0%) to 2015 (20.0%). This increasing time trend is consistent with findings of a retrospective study in adults with AN, in which antipsychotic use doubled from 1997–2002 (8.9%) to 2003–2009 (18.5%) [27].

Olanzapine was the most frequently used antipsychotic not only in the present study (64%) but also across all retrospective studies in youth published to date, ranging from 6 to 69% [45, 46, 56], except for one retrospective chart review in youth with AN (48%), Bulimia nervosa (BN) (5%) and Other specified feeding and eating disorders (OSFED) (47%), which found quetiapine to be the most frequent (75%) and olanzapine the second most frequent (21.9%) antipsychotic medication [57]. So far, only three studies in pediatric AN patients focused on non-olanzapine antipsychotics, i.e., aripiprazole [47], risperidone [50], and quetiapine [58]. In our study, in 34% of the 44 patients with AN receiving antipsychotics (29% of the 52 antipsychotic trials), the antipsychotic was stopped due to various reasons, which is consistent with the drop-out rate of 28% of ten randomized and open studies of antipsychotics in youth and adults with AN included in a recent meta-analysis [35].

In multivariable logistic regression analyses, antipsychotic use was associated with younger age at admission, more frequent history of childhood abuse, comorbid borderline personality disorder traits, and less weight change/week before antipsychotic administration. Patients treated with antipsychotics had lower weight and weight change parameters at discharge and received significantly more often other psychotropic medications than patients not receiving antipsychotics, indicating that antipsychotics were given to a more severely ill subgroup of AN patients. These findings are consistent with the first retrospective chart review in 43 youth with AN treated with olanzapine that reported more comorbidities, a lower BMI at admission, a longer treatment duration, greater rates of specific eating disorder treatment settings (i.e., day-hospital or inpatient treatment), and subsequent readmission [46]. A second retrospective chart review of 22 adolescents also found more psychiatric comorbidities in patients treated with aripiprazole [47], while a third retrospective study found in 604 youth (age = 15.3 ± 2.3 years, 91% females) that only prior hospitalization was correlated with antipsychotic use [19]. Finally, a fourth retrospective chart review of 86 patients with EDs (75% with AN, mean age = 16, range = 8–24 years) reported an association of a longer duration of illness and history of non-suicidal self-injury, but not of psychiatric comorbidities, with antipsychotics and antidepressant use [56]. However, this study did not differentiate between antipsychotics and antidepressants in terms of associated factors.

The matched subsample 1 confirmed our findings of lower anthropometric measures at discharge and less favorable weight change parameters in the antipsychotic-treated group, indicating no significant benefits on weight change in youth with AN. Olanzapine, the most frequently used antipsychotic, also did not improve weight gain more than other antipsychotics in our study but was prescribed to patients with lower anthropometric parameters at admission, i.e., to more severely ill inpatients. These negative findings matched on 14 measured variables likely mitigated or ruled out a selection by indication bias and are consistent with all currently available meta-analyses of randomized controlled trials conducted in mostly adult samples with AN, except for one (see below) that also found no significant benefits of antipsychotics on weight gain in AN [20, 26, 34–37]. Nevertheless, there are few single RCTs and chart review studies that showed positive effects of antipsychotic medications on weight change in adult as well as pediatric patients with AN, most notably, the most recent and largest RCT that compared olanzapine versus placebo in 152 adult outpatients over 16 weeks [39]. Furthermore, the most recent meta-analysis, including this currently largest and positive RCT in adults with olanzapine [39], showed a significant weight gain in adults for olanzapine versus placebo in adults, but not for olanzapine as adjuvant treatment in adolescents [40]. Individual, smaller and shorter RCTs in adults had also shown either greater and more rapid weight gain (n = 34, 100% females, day hospital, 13 weeks, olanzapine vs. placebo) [59] or increased BMI (in AN-BP) and improvement of psychopathology (n = 30, 100% females, outpatients, 12 weeks, cognitive behavioral therapy (CBT) plus olanzapine versus CBT plus placebo [60].

However, in youth, there are even fewer and smaller RCTs available that all have shown no significant effects on weight change and AN-related cognitions: One small RCT (n = 20, age = 12–21 years) showed no evidence in weight gain, eating attitudes, and behaviors or psychological functioning for olanzapine versus placebo [49]. The only RCT on risperidone versus placebo in youth with AN so far (n = 40, age = 12–21 years, mean age = 16) also did not find a significant effect on weight gain or typical anorexia-related cognitions (drive for thinness, body dissatisfaction, body image distortion) [50]. The only RCT comparing treatment as usual (TAU) plus quetiapine with TAU in a mixed sample of adolescents and adults (n = 33, age 12–42 years, mean age = 22) did not show significant differences in psychometric outcomes and weight gain between the groups [58]. To date, there is only one meta-analysis in adolescents (3 studies, n = 65) that examined the effect of olanzapine as an adjuvant treatment which did not find a significant weight gain in this age group [40].

Reasons for prescribing antipsychotic medication to youth with AN, despite the current lack of evidence supporting antipsychotic efficacy for body weight gain and AN-related cognitions, are likely due to the fact that these patients are considered relatively refractory to therapy as seen by lower weight change parameters before antipsychotic medication initiation, prompting actions in hopes of improving outcomes. The mismatch between evidence and practice calls for further research in larger samples to rule out the lack of significant effects due to small samples and to identify subgroups of youth who may benefit most from antipsychotic use, promoting stratified or, even, personalized care.

The results of this study need to be interpreted within its limitations. First, RCTs are more reliable than retrospective cohort studies; thus, all results of the present study have to be viewed with caution. In order to mitigate against confounding by indication, i.e., poorer outcomes being predicted by a selection of patients for antipsychotic use who are inherently less responsive to AN treatment, we repeated the analyses with a control sample of youth not receiving antipsychotics that were matched on 14 relevant baseline parameters. Nevertheless, there could have been residual confounding due to unmeasured variables, notably AN-specific psychopathology. Second, despite the overall large sample, the sample with versus without antipsychotic use (n = 44 vs. n = 250) was unbalanced and the subgroup of antipsychotic-treated youth was still modest. Additionally, we were only able to analyze those patients who agreed to antipsychotic medication use and not all patients who were offered antipsychotics. A considerable group of patients with AN refuse antipsychotic medication because they fear the potential of weight gain [27, 50, 61]. However, despite this selection bias, we were able to analyze the true effect of antipsychotic use, given in an inpatient setting with little opportunity to non-adherence, using an objective outcome measure, body weight. Third, the antipsychotics used included predominantly olanzapine (63%), and data on more recently approved antipsychotics, such as aripiprazole and cariprazine, were missing. Fourth, while a subanalysis of patients with AN-R versus those with AN-BP would have been of interest, the sample size was too small to conduct such analyses. However, future studies with larger and carefully matched samples of youth with AN receiving versus not receiving antipsychotics should explore potential differences in the results by AN-subtype. Fifth, the total and especially the antipsychotic sample both had a large percentage of patients with antipsychotic and additional medication (19% of the total sample (14% without antipsychotic use, 46% with antipsychotic use) received antidepressant cotreatment, mainly SSRIs). However, only 6 patients (7%) in the matched subsample received paroxetine (= 1) or mirtazapine (n = 5) which could lead to weight gain. Moreover, all of these patients were in the antipsychotic-treated sample, which could have biased the results toward a more favorable weight gain trajectory in the antipsychotic-treated sample, a finding that we were unable to confirm. Nevertheless, it is possible that the underlying comorbidities that gave rise to antidepressant use may have affected the outcome, despite careful matching on at least one psychiatric comorbidity, major depressive disorder, and borderline personality disorder traits (*p* > 0.8). Sixth, the analyzed sample covered a long period of time. Hence, we are not able to rule out a potential time effect regarding the change in the non-pharmacologic treatment program over the years. Seventh, only information collected as part of standard care was available for analysis and matching. Information was not available on AN-related psychopathology, such as fear of being “fat” and body image distortion, eating behaviors, (hyper)activity levels, depressive, anxious, or obsessive-compulsive symptoms, and type and intensity of psychosocial interventions. Such information is obviously very important and should be collected in further studies. Finally, side effects of antipsychotics, including cardiometabolic parameters, were not recorded systematically, and we were unable to classify the reasons that led to changing or ending antipsychotic treatment, which could have included tolerability issues. Such information should be collected in future studies more systematically.

Nevertheless, despite these limitations, to our knowledge, this is the currently largest retrospective chart review of pediatric inpatients with AN reporting on the characteristics, correlates, and effects of antipsychotics on weight gain. Further, to the best of our knowledge, the present study, including 13 years of data on antipsychotic use, also covers the longest time period of the available retrospective cohort studies of youth with AN receiving antipsychotic medications, with the other studies ranging from 2 years [56, 57] to 3 years [47], 4 years [45], and up to 6 years [46]. Additionally, we examined and were able to likely rule out several potential confounders, such as selection bias, confounding by indication bias, and bias by including less weight-gain producing, non-olanzapine antipsychotics, utilizing a stringent and extensive matching protocol.

In summary, in this naturalistic study in 294 pediatric inpatients with AN, antipsychotics, which were given preferentially to a more severely ill subgroup, failed to show benefits on weight gain, both across and within group, including after careful matching. Thus, despite absent evidence and contrary guidelines recommendations, antipsychotic medication is given to a relevant subgroup of youth with AN. Therefore, future research is essential and should include RCTs and naturalistic studies in youth, especially on the newer and well-tolerated antipsychotic medications, such as aripiprazole. Such studies should include information on AN-related psychopathology, type, and severity of psychiatric comorbidities, type and intensity of psychosocial interventions, as well as detailed adverse effects of antipsychotics, including metabolic parameters. Until such data are available, clinicians need to balance the unknown benefits with potential risks of antipsychotic use in youth with AN, monitoring both efficacy and safety carefully to justify the ongoing use of antipsychotics in youth with AN.

### Supplementary Information

**Additional file 1.** Supplemental Material, Tables and Figures.

## Data Availability

The dataset generated and analyzed during the current study is not publicly available because of privacy and ethical restrictions due to the high data protection of children and adolescents' data in psychiatric institutions in Germany. However, they are available from the corresponding author on reasonable request.

## Abbreviations

AN: Anorexia nervosa
AN-A: Anorexia nervosa, atypical subtype
AN-BP: Anorexia nervosa, binge-purging subtype
AN-R: Anorexia nervosa, restricting subtype
ARFID: Avoidant/restrictive food intake disorder
BMI: Body mass index
BN: Bulimia nervosa
CBT: Cognitive behavioral therapy
ED: Eating disorder
FGA: First-generation antipsychotic
ICD-10: International Classification of Diseases, 10th revision
IQR: Interquartile range
MDD: Major depressive disorder
NGT: Nasogastric tube
OSFED: Other specified feeding and eating disorders
RCT: Randomized controlled study
SGA: Second-generation antipsychotic
SPSS: Statistical package for social sciences
SSRI: Selective serotonin reuptake inhibitor
TAU: Treatment as usual
