# Supplementary material for: Antipsychotic effects on anthropometric outcomes in anorexia nervosa: a retrospective chart review of hospitalized children and adolescents
Source: J Eat Disord. 2023 Sep 6;11:151. doi: 10.1186/s40337-023-00862-4 (PMC10481458; doi:10.1186/s40337-023-00862-4)
Supplement: Supplementary file 1 — Additional file 1. Supplemental Material, Tables and Figures. [file 40337_2023_862_MOESM1_ESM.docx]

**Supplemental Material**

**Supplementary Table 1. Weight change characteristics of anorexia nervosa patients with olanzapine vs. anorexia nervosa patients with other antipsychotics**

|  | **Total**  **(n=44)** | | **With**  **Olanzapine**  **(n=33)** | | **With other antipsychotics**  **(n=11)** | | **Mann-Whitney U-test** | | | **Cramers V** |
| --- | --- | --- | --- | --- | --- | --- | --- | --- | --- | --- |
|  | **Mdn** | **(Q1,Q3)** | **Mdn** | **(Q1,Q3)** | **Mdn** | **(Q1,Q3)** | **Z** | **U** | **p** |  |
| **Anthropometric Characteristics at Baseline** | | | | | | | | | | |
| BMI at admission | 14.4 | (13.4,15.0) | 14.4 | (13.5,15.0) | 14.7 | (13.2,15.5) | -1.0 | 144.0 | 0.309 | 0.2 |
| BMI percentile at admission | 1.0 | (1.0,1.0) | 1.0 | (1.0,1.0) | 1.0 | (1.0,5.0) | -2.8 | 112.5 | 0.005* | 0.4 |
| BMI z-scores at admission | -2.8 | (-3.7,-2.4) | -3.0 | (-3.8,-2.4) | -2.3 | (-3.5,-1.6) | -2.1 | 104.5 | 0.037* | 0.3 |
| Kg < 1. BMI percentile at admission | -1.8 | (-3.6,0.0) | -2.1 | (-3.8,-0.2) | 0.0 | (-2.7,0.0) | -2.0 | 108.0 | 0.043* | 0.3 |
| **Anthropometric Characteristics at Discharge** | | | | | | | | | | |
| BMI at discharge | 17.0 | (16.0,17.5) | 17.0 | (16.2,17.7) | 16.8 | (16.0,17.2) | -0.8 | 147.0 | 0.419 | 0.1 |
| BMI percentile at discharge | 9.0 | (3.0,14.0) | 8.0 | (2.3,14.0) | 13.0 | (3.0,18.0) | -0.9 | 145.5 | 0.395 | 0.1 |
| BMI z-scores at discharge | -1.4 | (-2.0,-1.1) | -1.5 | (-2.0,-1.1) | -1.1 | (-1.9,-0.9) | -1.1 | 138.0 | 0.290 | 0.2 |
| Weight change, kg/week | 0.4 | (0.3,0.6) | 0.4 | (0.3,0.6) | 0.3 | (0.3,0.4) | -1.1 | 138.0 | 0.290 | 0.2 |
| BMI change/week | 0.1 | (0.1,0.2) | 0.1 | (0.1,0.3) | 0.1 | (0.1,0.2) | -1.1 | 136.0 | 0.266 | 0.2 |
| BMI percentiles change/week | 0.3 | (0.1,0.9) | 0.3 | (0.0,0.7) | 0.8 | (0.2,0.9) | -0.9 | 144.5 | 0.379 | 0.1 |
| BMI z-score change/week | 0.1 | (0.1,0.1) | 0.1 | (0.1,0.1) | 0.1 | (0.1,0.1) | -0.8 | 148.0 | 0.436 | 0.1 |

*Note*. IQR=interquartile range, Mdn=median, ** p<0.05*

**Supplementary Table 2. Weight change characteristics of anorexia nervosa patients with antipsychotics after hospital discharge vs. no antipsychotics after hospital discharge**

|  | **Total**  **(n=44)** | | **Antipsychotics after discharge**  **(n=26)** | | **No antipsychotics after discharge**  **(n=18)** | | **Mann-Whitney U-test** | | | **Cramers V**  **Cohens d** |
| --- | --- | --- | --- | --- | --- | --- | --- | --- | --- | --- |
|  | **Mdn** | **(Q1,Q3)** | **Mdn** | **(Q1,Q3)** | **Mdn** | **(Q1,Q3)** | **Z** | **U** | **p** |  |
| **Anthropometric Characteristics at Baseline** | | | | | | | | | | |
| BMI at admission | 14.4 | (13.4,15.0) | 14.5 | (13.5,15.1) | 14.3 | (13.2,14.7) | -0.9 | 197.5 | 0.383 | 0.1 |
| BMI percentile at admission | 1.0 | (1.0,1.0) | 1.0 | (1.0,1.0) | 1.0 | (1.0,1.0) | -1.2 | 200.0 | 0.227 | 0.2 |
| BMI z-scores at admission | -2.8 | (-3.7,-2.4) | -2.7 | (-3.7,-2.3) | -3.1 | (-3.8,-2.3) | -0.8 | 199.0 | 0.403 | 0.1 |
| Kg < 1. BMI percentile at admission | -1.8 | (-3.6,0.0) | -1.2 | (-3.0,0.0) | -2.1 | (4.1,0.0) | -0.7 | 205.5 | 0.491 | 0.1 |
| **Anthropometric Characteristics at Discharge** | | | | | | | | | | |
| BMI at discharge | 17.0 | (16.0,17.5) | 17.0 | (16.0,17.4) | 17.0 | (16.3,18.0) | -0.8 | 193.5 | 0.438 | 0.1 |
| BMI percentile at discharge | 9.0 | (3.0, 14.0) | 7.0 | (3.0,15.0) | 11.0 | (4.3,14.3) | -0.6 | 201.5 | 0.562 | 0.1 |
| BMI z-scores at discharge | -1.4 | (-2.0,-1.1) | -1.5 | (-2.0,-1.0) | -1.3 | (-1.7,-1.1) | -0.6 | 200.5 | 0.546 | 0.1 |
| Weight change, kg/week | 0.4 | (0.3,0.6) | 0.4 | (0.3,0.6) | 0.4 | (0.2,0.6) | -0.3 | 212.0 | 0.749 | 0.0 |
| BMI change/week | 0.1 | (0.1,0.2) | 0.1 | (0.1,0.2) | 0.2 | (0.1,0.2) | -0.5 | 203.0 | 0.588 | 0.1 |
| BMI percentiles change/week | 0.3 | (0.1,0.9) | 0.3 | (0.0, 0.8) | 0.4 | (0.2,0.9) | -0.6 | 200.5 | 0.545 | 0.1 |
| BMI z-score change/week | 0.1 | (0.1,0.1) | 0.1 | (0.1,0.1) | 0.1 | (0.1,0.1) | -0.3 | 213.5 | 0.777 | 0.0 |

*Note*. IQR=interquartile range, Mdn=median, ** p<0.05*

**Supplementary Table 3. Clinical characteristics of anorexia nervosa patients with vs. without antipsychotic medication - matched sample 2**

|  | **Total**  **(n=62)** | | **With**  **antipsychotics**  **(n=31)** | | **Without**  **Antipsychotics**  **(n=31)** | | **t-test**  **Mann-Whitney U-test**  **χ^2^-test** | | | **Cohens d**  **r** |
| --- | --- | --- | --- | --- | --- | --- | --- | --- | --- | --- |
|  | **M**  **Mdn**  **n** | **± SD**  **(Q1,Q3)**  **(%)** | **M**  **Mdn**  **n** | **± SD**  **(Q1,Q3)**  **(%)** | **M**  **Mdn**  **n** | **± SD**  **(Q1,Q3)**  **(%)** | **df**  **z** | **t**  **U**  **χ^2^** | **p** |  |
| ED diagnoses | - |  | - |  | - |  | 1 | 0.1 | 0.776 | 0.0 |
| AN-R | 45 | (73) | 23 | (74) | 22 | (71) | - | - | - | - |
| AN-BP | 17 | (27) | 8 | (26) | 9 | (29) | - | - | - | - |
| Sex | - |  | - |  | - |  | 1 | 0.0 | 1.000 | 0.0 |
| Female | 60 | (97) | 30 | (97) | 30 | (97) | - | - | - | - |
| Male | 2 | (3) | 1 | (3) | 1 | (3) | - | - | - | - |
| Age of onset, years | 13.6 | (12.7,15.0) | 13.7 | (12.4,15.0) | 13.2 | (12.7,15.1) | -0.3 | 459.5 | 0.767 | 0.1 |
| Duration of illness, months | 11.2 | ± 1.2 | 12.0 | ± 1.9 | 10.5 | ± 1.4 | 56.0 | -0.6 | 0.528 | 0.2 |
| Age at admission, years | 14.5 | (13.5,16.2) | 14.6 | (13.1,16.2) | 14.5 | (13.8,16.4) | -0.2 | 464.0 | 0.816 | 0.1 |
| History of childhood emotional abuse | 5 | (8) | 3 | (10) | 2 | (7) | 1 | 0.2 | 0.641 | 0.1 |
| History of childhood physical abuse | 7 | (11) | 5 | (16) | 2 | (7)1 | 1 | 1.4 | 0.229 | 0.2 |
| Comorbidities, average number | 1.0 | (0.0,1.0) | 0.0 | (0.0,1.0) | 0.0 | (0.0,1.0) | -0.3 | 461.0 | 0.767 | 0.1 |
| At least one comorbidity | 38 | (61) | 20 | (65) | 18 | (58) | 1 | 0.3 | 0.602 | 0.1 |
| Major depressive disorder | 22 | (36) | 11 | (36) | 11 | (36) | 1 | 0.0 | 1.000 | 0.0 |
| Borderline personality disorder traits | 4 | (7) | 3 | (10) | 1 | (3) | 1 | 1.1 | 0.301 | 0.1 |
| **Anthropometric Characteristics at Baseline** | | | | | | | | | | |
| BMI at admission | 14.2 | (13.4,15.0) | 14.4 | (13.1,15.0) | 14.2 | (13.5,15.2) | -0.3 | 460.5 | 0.778 | 0.1 |
| BMI percentile at admission | 1.0 | (1.0,1.0) | 1.0 | (1.0,1.0) | 1.0 | (1.0,1.0) | -0.4 | 464.0 | 0.672 | 0.1 |
| BMI z-scores at admission | -3.0 | (-3.7,-2.5) | -3.0 | (-3.8,-2.5) | -3.1 | (-3.5,-2.5) | -0.2 | 465.0 | 0.827 | 0.1 |
| Kg < 1. BMI percentile at admission | -2.0 | (-3.6,-0.1) | -2.0 | (-3.6,-0.1) | -2.0 | (-3.6,0.0)1 | -0.2 | 469.0 | 0.870 | 0.0 |
| **Anthropometric Characteristics at Discharge** | | | | | | | | | | |
| BMI at discharge | 17.0 | (16.4,17.8) | 16.8 | (16.0,17.6) | 17.2 | (16.4,18.4) | -1.8 | 354.5 | 0.076 | 0.2 |
| BMI percentile at discharge | 8.5 | (4.0,16.0) | 7.0 | (2.0,14.0) | 11.0 | (6.0,18.0) | -2.1 | 333.5 | 0.038* | 0.5 |
| BMI z-scores at discharge | -1.4 | (-1.8,-1.0) | -1.5 | (-2.1,-1.1) | -1.2 | (-1.6,-0.9) | -2.3 | 316.0 | 0.021* | 0.6 |
| Weight change, kg/week | 0.4 | (0.3,0.5) | 0.3 | (0.3,0.5) | 0.5 | (0.3,0.6) | -2.2 | 324.0 | 0.028* | 0.6 |
| BMI change/week | 0.2 | (0.1,0.2) | 0.1 | (0.1,0.2) | 0.2 | (0.1,0.2) | -1.4 | 380.0 | 0.157 | 0.4 |
| BMI percentiles change/week | 0.4 | (0.2,0.9) | 0.3 | (0.0,0.7) | 0.5 | (0.3,1.0) | -2.3 | 317.5 | 0.022* | 0.6 |
| BMI z-score change/week | 0.1 | (0.1,0.1) | 0.1 | (0.1,0.1) | 0.1 | (0.1,0.1) | -1.5 | 374.0 | 0.134 | 0.4 |
| **Psychotropic medication other than antipsychotics during hospitalization** | | | | | | | | | | |
| Psychotropic medication other than antipsychotics | 22 | (35.5) | 12 | (38.7) | 10 | (32.3) | 1 | 0.3 | 0.596 | 0.1 |
| One | 16 | (25.8) | 7 | (22.6) | 9 | (29.0) | - | - | - | - |
| Two | 4 | (6.5) | 3 | (9.7) | 1 | (3.2) | - | - | - | - |
| Three | 2 | (3.2) | 2 | (6.5) | 0 | (0.0) | - | - | - | - |
| Four | 0 | (0.0) | 0 | (0.0) | 0 | (0.0) | - | - | - | - |
| Antidepressants, average number | 0.0 | (0.0,1.0) | 0.0 | (0.0,1.0) | 0.0 | (0.0,1.0) | -0.8 | 434.5 | 0.440 | 0.2 |
| **At least one antidepressant** | 22 | (35.5) | 12 | (38.7) | 10 | (32.3) | 1 | 0.3 | 0.596 | 0.1 |
| **SSNRI** | 2 | (3.2) | 1 | (3.2) | 1 | (3.2) | 1 | 0.0 | 1.000 | 0.0 |
| Venlafaxine | 2 | (3.2) | 1 | (3.2) | 1 | (3.2) | 1 | 0.0 | 1.000 | 0.0 |
| **SSRI, at least one** | 19 | (30.6) | 10 | (32.3) | 9 | (29.0) | 1 | 0.0 | 1.000 | 0.0 |
| Escitalopram | 12 | (19.4) | 5 | (16.1) | 7 | (22.6) | 1 | 0.1 | 0.748 | 0.1 |
| Fluoxetine | 3 | (4.8) | 2 | (6.5) | 1 | (3.2) | 1 | 0.0 | 1.000 | 0.1 |
| Fluvoxamine | 3 | (4.8) | 2 | (6.5) | 1 | (3.2) | 1 | 0.0 | 1.000 | 0.1 |
| Paroxetine | 1 | (1.6) | 1 | (3.2) | 0 | (0.0) | 1 | 0.0 | 1.000 | 0.1 |
| **Tetracyclic antidepressant** | 4 | (6.5) | 4 | (12.9) | 0 | (0.0) | 1 | 2.4 | 0.113 | 0.3 |
| Mirtazapine | 4 | (6.5) | 4 | (12.9) | 0 | (0.0) | 1 | 2.4 | 0.113 | 0.3 |
| **Tricyclic antidepressant** | 2 | (3.2) | 1 | (3.2) | 1 | (3.2) | 1 | 0.0 | 1.000 | 0.0 |
| Doxepin | 2 | (3.2) | 1 | (3.2) | 1 | (3.2) | 1 | 0.0 | 1.000 | 0.0 |
| **Anxiolytic** | 2 | (3.2) | 2 | (6.5) | 0 | (0.0) | 1 | 0.5 | 0.472 | 0.2 |
| Lorazepam | 2 | (3.2) | 2 | (6.5) | 0 | (0.0) | 1 | 0.5 | 0.472 | 0.2 |

*Note*. AN-BP=anorexia nervosa, binge-purge type, AN-R=AN, restricting type, BN=bulimia nervosa, IQR=interquartile range, Mdn=median, SD=standard deviation; artificial median split at treatment week 5 for the group without antipsychotics, ** p<0.05*

**Supplemental Table 4. Clinical characteristics of anorexia nervosa patients with antipsychotic medication with a fast vs. slower* weekly weight change**

|  | | **Total**  **(n=43)** | | | **Faster weekly**  **weight change**  **(n=21)** | | | **Slower weekly weight change**  **(n=22)** | | | | **t-test**  **Mann-Whitney U-test**  **χ^2^-test** | | | | | |  | | |
| --- | --- | --- | --- | --- | --- | --- | --- | --- | --- | --- | --- | --- | --- | --- | --- | --- | --- | --- | --- | --- |
|  | | **M**  **Mdn**  **n** | **± SD (Q1,Q3)**  **(%)** | | **M**  **Mdn**  **n** | **± SD**  **(Q1,Q3)**  **(%)** | | **M**  **Mdn**  **n** | | **± SD**  **(Q1,Q3)**  **(%)** | | **df**  **z** | | **t**  **U**  **χ^2^** | | | **p** | **Cohens d**  **Cramers V** | | |
| ED diagnoses | |  | - | |  | - | |  | | - | | 1 | | 0.9 | | | 0.337 | 0.2 | | |
| AN-R | | 32 | (74) | | 17 | (81) | | 15 | | (68) | | - | | - | | | - | - | | |
| AN-BP | | 11 | (26) | | 4 | (19) | | 7 | | (32) | | - | | - | | | - | - | | |
| Sex | |  | - | |  | - | |  | | - | | 1 | | 1.1 | | | 0.300 | 0.2 | | |
| Female | | 42 | (98) | | 20 | (95) | | 22 | | (100) | | - | | - | | | - | - | | |
| Male | | 1 | (2) | | 1 | (5) | | 0 | | (0) | | - | | - | | | - | - | | |
| Age of onset, years | | 13.6 | (12.2,14.4) | | 13.7 | (12.4,14.7) | | 13.6 | | (12.1,14.0) | | -0.7 | | 203.0 | | | 0.496 | 0.2 | | |
| Duration of illness, months | | 13.8 | ± 1.9 | | 11.5 | ± 1.9 | | 14.8 | | ± 3.0 | | 35.5 | | 0.9 | | | 0.363 | 0.3 | | |
| Age at admission, years | | 14.8 | (13.2,16.0) | | 14.9 | (13.8,15.9) | | 14.5 | | (12.9,16.2) | | -0.3 | | 217.0 | | | 0.734 | 0.1 | | |
| Menarche, age in years | | 12.0 | (11.0,13.0) | | 12.0 | (11.5,12.5) | | 13.0 | | (11.0,13.0) | | -0.5 | | 43.0 | | | 0.609 | 0.2 | | |
| Primary amenorrhea | | 13 | (30) | | 6 | (29) | | 7 | | (32) | | 0.1 | | 1 | | | 0.817 | 0.0 | | |
| Secondary amenorrhea | | 23 | (54) | | 12 | (57) | | 11 | | (50) | | 0.2 | | 1 | | | 0.639 | 0.1 | | |
| Oligomenorrhea | | 1 | (2) | | 0 | (0) | | 1 | | (5) | | 1.0 | | 1 | | | 0.323 | 0.2 | | |
| **Intelligence** | | | | | | | | | | | | | | | | | |  | | |
| Very high intelligence | | 2 | (5) | | 1 | (5) | | 1 | | (5) | | 0.0 | | 1 | | | 0.973 | 0.0 | | |
| High intelligence | | 19 | (44) | | 8 | (38) | | 11 | | (50) | | 0.6 | | 1 | | | 0.432 | 0.1 | | |
| Average intelligence | | 21 | (49) | | 11 | (52) | | 10 | | (46) | | 0.2 | | 1 | | | 0.650 | 0.1 | | |
| Below average intelligence | | 1 | (3) | | 1 | (5) | | 0 | | (0) | | 1.1 | | 1 | | | 0.300 | 0.2 | | |
| **Family psychopathology and childhood abuse** | | | | | | | | | | | | | | | | | |  | | |
| Family psychopathology present | | 31 | (72) | | 11 | (52) | | 20 | | (91) | | 7.9 | | 1 | | | 0.005* | 0.4 | | |
| History of childhood abuse | | 9 | (21) | | 3 | (14) | | 6 | | (27) | | 1.1 | | 1 | | | 0.295 | 0.2 | | |
| Emotional | | 5 | (12) | | 3 | (14) | | 2 | | (9) | | 0.3 | | 1 | | | 0.595 | 0.1 | | |
| Physical | | 5 | (12) | | 1 | (5) | | 4 | | (18) | | 1.9 | | 1 | | | 0.170 | 0.2 | | |
| Sexual | | 2 | (5) | | 0 | (0) | | 2 | | (9) | | 2.0 | | 1 | | | 0.157 | 0.2 | | |
| **Psychiatric comorbidities** | | | | | | | | | | | | | | | | | |  | | |
| Comorbidities, average number | | 1.0 | (0.0,1.0) | | 1.0 | (0.0,1.0) | | 1.0 | | (0.0,1.0) | | -0.7 | | 205.0 | | | 0.495 | 0.2 | | |
| At least one comorbidity | | 28 | (65) | | 12 | (57) | | 16 | | (73) | | 1.1 | | 1 | | | 0.284 | 0.2 | | |
| Substance use disorder | | 1 | (3) | | 1 | (5) | | 0 | | (0) | | 1.1 | | 1 | | | 0.300 | 0.2 | | |
| Affective disorders | | 21 | (49) | | 8 | (38) | | 13 | | (59) | | 1.9 | | 1 | | | 0.169 | 0.2 | | |
| Major depressive disorder | | 8 | (19) | | 3 | (14) | | 5 | | (23) | | 0.5 | | 1 | | | 0.477 | 0.1 | | |
| Persistent affective disorder | | 14 | (33) | | 5 | (24) | | 9 | | (41) | | 1.4 | | 1 | | | 0.232 | 0.2 | | |
| Neurotic, stress, and somatoform disorders | | 8 | (19) | | 5 | (24) | | 3 | | (14) | | 0.7 | | 1 | | | 0.391 | 0.1 | | |
| Phobia | | 1 | (2) | | 0 | (0) | | 1 | | (5) | | 1.0 | | 1 | | | 0.323 | 0.2 | | |
| Obsessive-compulsive disorder | | 6 | (14) | | 4 | (19) | | 2 | | (9) | | 0.9 | | 1 | | | 0.346 | 0.1 | | |
| Post-traumatic stress disorder | | 0 | (0) | | 0 | (0) | | 0 | | (0) | | - | | - | | | - | - | | |
| Personality and behavioral disorder traits | | 6 | (14) | | 2 | (10) | | 4 | | (18) | | 0.7 | | 1 | | | 0.413 | 0.1 | | |
| Borderline | | 3 | (7) | | 1 | (5) | | 2 | | (9) | | 0.3 | | 1 | | | 0.578 | 0.1 | | |
| Compulsive | | 1 | (2) | | 1 | (5) | | 0 | | (0) | | 1.1 | | 1 | | | 0.300 | 0.2 | | |
| Disorders with onset in childhood/adolescence | | 0 | (0) | | 0 | (0) | | 0 | | (0) | | - | | - | | | - | - | | |
| **Anthropometric Characteristics at Baseline** | | | | | | | | | | | | | | | | | | | | |
| BMI at admission | | 14.4 | (13.4,15.0) | | 14.1 | (13.1,15.0) | | 14.4 | | (13.5,15.1) | | -0.6 | | 207.5 | | | 0.568 | 0.2 | | |
| BMI percentile at admission | | 1.0 | (1.0,1.0) | | 1.0 | (1.0,1.0) | | 1.0 | | (1.0,1.0) | | -1.5 | | 190.0 | | | 0.142 | 0.3 | | |
| BMI z-scores at admission | | -2.8 | (-3.7,-2.4) | | -3.0 | (-4.0,-2.5) | | -2.8 | | (-3.6,-2.1) | | -1.1 | | 184.0 | | | 0.253 | 0.4 | | |
| Kg < 1. BMI percentile at admission | | -1.8 | (-3.6,0.0) | | -2.1 | (-4.2,-0.1) | | -0.9 | | (-3.0,0.0) | | -1,5 | | 170.0 | | | 0.133 | 0.5 | | |
| **Anthropometric Characteristics at Discharge** | | | | | | | | | | | | | | | | | | | | |
| BMI at discharge | | 16.9 | (16.0,17.5) | | 17.3 | (16.7,18.0) | | 16.5 | | (15.8,17.0) | | -2.6 | | 123.0 | | | 0.009* | 0.9 | | |
| BMI percentile at discharge | | 9.0 | (3.0,14.0) | | 13.0 | (6.5,16.5) | | 5.5 | | (2.0,12.5) | | -2.0 | | 147.5 | | | 0.042* | 0.7 | | |
| BMI z-scores at discharge | | -1.4 | (-2.0,-1.1) | | -1.2 | (-1.6,-1.0) | | -1.6 | | (-2.1,-1.1) | | -1.7 | | 162.0 | | | 0.094 | 0.5 | | |
| **Treatment duration, days** | 124.1 | | | ± 8.4 | 101.1 | ± 6.4 | 142.1 | | | | ± 13.9 | | 29.4 | | -2.7 | 0.012* | | | -0.8 |  |
| **Antipsychotic medications during hospitalization** | | | | | | | | | | | | | | | | | | | |  |
| Yes | 43 | | | (100) | 21 | (100) | 22 | | | | (100) | | - | | - | - | | | - |  |
| Duration (weeks) | 6.7 | | | ± 3.9 | 6.1 | ± 4.5 | 4.5 | | | | ± 1.0 | | 39 | | 0.8 | 0.425 | | | 0.3 |  |
| **Psychotropic medication other than antipsychotics** | | | | | | | | | | | | | | | | | | | |  |
| Psychotropic medication other than antipsychotics | 19 | | | (44.2) | 8 | (38.1) | 11 | | (50.0) | | | | 1 | | 0.2 | 0.632 | | | 0.1 |  |
| One | 14 | | | (32.6) | 8 | (38.1) | 6 | | (27.3) | | | | 1 | | - | - | | | - |  |
| Two | 3 | | | (7.0) | 0 | (0.0) | 3 | | (13.6) | | | | 1 | | - | - | | | - |  |
| Three | 2 | | | (4.7) | 0 | (0.0) | 2 | | (9.1) | | | | 1 | | - | - | | | - |  |
| Four | 0 | | | (0.0) | 0 | (0.0) | 0 | | (0.0) | | | | 1 | | - | - | | | - |  |
| Antidepressants, average number | 0.0 | | | (0.0,1.0) | 0.0 | (0.0,1.0) | 0.0 | | (0.0,1.0) | | | | -0.8 | | 203.5 | 0.437 | | | 0.2 |  |
| **At least one antidepressant** | 19 | | | (44.2) | 8 | (38.1) | 11 | | (50.0) | | | | 1 | | 0.2 | 0.632 | | | 0.1 |  |
| **SSNRI** | 1 | | | (2.3) | 0 | (0.0) | 1 | | (4.5) | | | | 1 | | 0.0 | 1.000 | | | 0.2 |  |
| Venlafaxine | 1 | | | (2.3) | 0 | (0.0) | 1 | | (4.5) | | | | 1 | | 0.0 | 1.000 | | | 0.2 |  |
| **SSRI, at least one** | 16 | | | (37.2) | 7 | (33.3) | 9 | | (40.9) | | | | 1 | | 0.0 | 0.843 | | | 0.1 |  |
| Escitalopram | 8 | | | (18.6) | 2 | (9.5) | 6 | | (27.3) | | | | 1 | | 1.2 | 0.240 | | | 0.2 |  |
| Fluoxetine | 5 | | | (11.6) | 3 | (14.3) | 2 | | (9.1) | | | | 1 | | 0.0 | 0.664 | | | 0.1 |  |
| Fluvoxamine | 2 | | | (4.7) | 2 | (9.5) | 0 | | (0.0) | | | | 1 | | 0.6 | 0.233 | | | 0.2 |  |
| Paroxetine | 1 | | | (2.3) | 0 | (0.0) | 1 | | (4.5) | | | | 1 | | 0.0 | 1.000 | | | 0.2 |  |
| **Tetracyclic antidepressant** | 5 | | | (11.6) | 0 | (0.0) | 5 | | (22.7) | | | | 1 | | 3.4 | 0.048* | | | 0.4 |  |
| Mirtazapine | 5 | | | (11.6) | 0 | (0.0) | 5 | | (22.7) | | | | 1 | | 3.4 | 0.048* | | | 0.4 |  |
| **Tricyclic antidepressant** | 1 | | | (2.3) | 1 | (4.8) | 0 | | (0.0) | | | | 1 | | 0.0 | 0.488 | | | 0.2 |  |
| Doxepin | 1 | | | (2.3) | 1 | (4.8) | 0 | | (0.0) | | | | 1 | | 0.0 | 0.488 | | | 0.2 |  |
| **Anxiolytic** | 2 | | | (4.7) | 0 | (0.0) | 2 | | (9.1) | | | | 1 | | 0.5 | 0.488 | | | 0.2 |  |
| Lorazepam | 2 | | | (4.7) | 0 | (0.0) | 2 | | (9.1) | | | | 1 | | 0.5 | 0.488 | | | 0.2 |  |

*Note*. AN-BP=anorexia nervosa, binge-purge type, AN-R=AN, restricting type, BN=bulimia nervosa, intelligence=very high (IQ>129), high (IQ 115-129), average (IQ 85-114), below average (IQ 70-84), IQR=interquartile range, Mdn=median, SD=standard deviation, ** p<0.05;* artificial median split at 0.354 kg weight change per week (slower weekly weight change <0.354 kg weight change per week vs. faster weekly weight change >0.354 kg weight change per week)

**Supplementary Figure 1. Weight change before, during, and after antipsychotics in patients with anorexia nervosa receiving antipsychotic medication (n=31)**

**Legend:**

**-- -- -- Weight change without antipsychotics, kg**

**--------- Weight change with antipsychotic, kg**

**- - - - - Weight change after antipsychotic, kg**
